# Allelic-Specific Regulation of xCT Expression Increases Susceptibility to Tuberculosis by Modulating microRNA-mRNA Interactions

**DOI:** 10.1128/mSphere.00263-20

**Published:** 2020-04-22

**Authors:** Wenfei Wang, Yi Cai, Guofang Deng, Qianting Yang, Peijun Tang, Meiying Wu, Ziqi Yu, Fan Yang, Jianyong Chen, Oliver Werz, Xinchun Chen

**Affiliations:** a Department of Pathogen Biology, Shenzhen University School of Medicine, Shenzhen, China; b Department of Pharmaceutical/Medicinal Chemistry, Institute of Pharmacy, Friedrich Schiller University, Jena, Germany; c Guangdong Key Laboratory for Emerging Infectious Diseases, Shenzhen Key Laboratory of Infection & Immunity, Shenzhen Third People’s Hospital, Shenzhen, China; d Department of Tuberculosis, The Fifth People’s Hospital of Suzhou, Suzhou, China; e Jiangxi Medical College, Nanchang University, Nanchang, China; University of Kentucky

**Keywords:** xCT, polymorphism, tuberculosis, genotype, therapy

## Abstract

xCT forms part of the x_c_^−^ cysteine-glutamate antiporter which inhibits antimicrobial inflammatory immune functions and thus increases susceptibility to tuberculosis (TB). However, the associations between xCT gene polymorphisms and susceptibility to TB, as well as whether these modulate xCT expression or affect treatment with the xCT inhibitor sulfasalazine (SASP), are unclear. In the present study, we genotyped xCT polymorphisms in a large Chinese cohort and found that the single-nucleotide polymorphism (SNP) rs13120371 was associated with susceptibility to TB. The rs13120371 AA genotype was strongly associated with an increased risk of TB and increased xCT mRNA expression levels compared to those with the GG or AG genotype. rs13120371 is located on the 3′ untranslated (UTR) region of the xCT gene, in the putative binding site for miR-142-3p, and the results of luciferase reporter assays indicated that the rs13120371 AA genotype inhibited the binding of miR-42-3p to xCT. Bacterial burden was also significantly higher in cells with the AA genotype than in those with the GG genotype. Furthermore, pretreatment with SASP alleviated this burden in cells with the AA genotype but conferred no benefit in cells with the GG phenotype. In summary, we identified a functional SNP (rs13120371) in the xCT 3′ UTR region that increases susceptibility to TB through interacting with miR-142-3p.

**IMPORTANCE** Tuberculosis (TB) is the leading cause of death from a single infectious agent globally, and the development of multidrug resistance represents a serious health concern, particularly in the developing world. Novel effective treatments are urgently required. xCT expression is known to increase susceptibility to TB, and certain polymorphisms in the gene encoding this protein interrupt the binding of microRNA and prevent its suppression. Taking advantage of the FDA approval for the use of sulfasalazine (SASP), which inhibits xCT-mediated cystine transport in humans, we demonstrate how host genotype-specific therapies tailored to the xCT genotype can improve TB outcomes.

## Abstract (precis)

Tuberculosis (TB) is the leading cause of death from a single infectious agent globally, and the development of multidrug resistance represents a serious health concern, particularly in the developing world. Novel effective treatments are urgently required. xCT expression is known to increase susceptibility to TB, and certain polymorphisms in the gene encoding this protein interrupt the binding of microRNA and prevent its suppression. Taking advantage of the FDA approval for the use of sulfasalazine (SASP), which inhibits xCT-mediated cystine transport in humans, we demonstrate how host genotype-specific therapies tailored to the xCT genotype can improve TB outcomes.

## INTRODUCTION

Tuberculosis (TB), a bacterial infection caused by Mycobacterium tuberculosis, is the leading cause of death from infectious disease globally (1). TB represents a major global health problem, claiming ∼1.7 million lives annually. An inadequate immune response to TB infection is thought to be linked to susceptibility to the disease (2–4), and host immunity and the inflammatory response are also closely linked with disease outcomes (5). Accordingly, certain genetic variants that affect the regulation of cell-mediated immune and inflammatory responses are also associated with susceptibility and host responses to TB (6–11). The differences in susceptibility to TB in particular ethnicities lends further credence to the role of genetic polymorphisms. Strong evidence suggests that polymorphisms in genes encoding Toll-like receptor (TLR), gamma interferon (IFN-γ), tumor necrosis factor alpha (TNF-α), interleukin 12 (IL-12), IL-10, monocyte chemoattractant protein 1 (MCP-1), matrix metalloproteinase 1 (MMP-1), and the vitamin D receptor (*VDR*) are involved (12–16). Further functional studies are required to fully characterize the impact of human genetic variation on TB and to identify additional candidate polymorphisms in genes that might be of interest. One such gene is *SLC7A11*, which encodes the protein xCT.

xCT is a subunit of the x_c_^−^ cysteine-glutamate antiporter: it is responsible for the Na^+^-independent transport of cystine into a cell in exchange for glutamate. This process is essential to cellular redox homeostasis, as it effectively increases the synthesis of glutathione, which serves as an antioxidant (17, 18). xCT is chaperoned by the CD98/Slc3a2 subunit, but unlike the constitutively expressed CD98, xCT expression is upregulated by oxidative stress. This is an important regulatory mechanism of the growth of both tumor cells and intracellular pathogens, such as M. tuberculosis. Accordingly, the genetic disruption of xCT and the chemical modulation of xCT with sulfasalazine (SASP), an inhibitor of xCT-mediated cystine transporters, have both been found to significantly decrease bactericidal burden and inflammation-related tissue damage in mice with TB (19).

SASP exerts inhibitory effects against tumor growth, invasion, and metastasis in many types of cancer (20, 21). As SASP has already received FDA approval for the treatment of ulcerative colitis, this gives it an advantage over other potential novel treatments for TB. However, the outcome of SASP treatment is still likely to be influenced by the genetics of the host. For example, a previous report demonstrated that a single-nucleotide polymorphism (SNP) in the human *LTA4H* promoter, rs17525495 TT, is associated with 2.3-fold higher LTA4H protein expression levels than the CC genotype. This SNP has been demonstrated to critically influence the response to anti-inflammatory dexamethasone treatment in TB meningitis (22, 23). This indicates that host genotype-specific therapies can optimize treatments for M. tuberculosis, as each patient can be matched to the treatment regimen that they are most likely to benefit from. However, for this strategy to have any hope of working, the impact of genetic polymorphisms on treatment response must be fully characterized.

It is clear that the identification of genetic variants that regulate xCT gene expression during M. tuberculosis infection are necessary if treatment of TB with SASP is to be successfully translated to the clinic. However, the associations between genetic polymorphisms in xCT and TB susceptibility remain largely unknown, and it is unclear how these variants modulate xCT expression or the effects of SASP treatment. To this end, we conducted a case-control study in a Chinese cohort to identify functional SNPs in the xCT gene and their links to TB susceptibility. We also investigated the effects and mechanisms underlying the effects of these functional SNPs on M. tuberculosis-induced xCT expression.

## RESULTS

### *xCT* gene polymorphism is associated with susceptibility to tuberculosis.

To analyze the relationship between xCT gene polymorphism and susceptibility to tuberculosis, four SNPs were screened with the MassARRAY platform, in a cohort of 914 active TB cases and 936 controls. Of these SNPs, rs4131888 and rs11764488 are located within the known intron region of the xCT gene, while rs7674870 and rs13120371 are located in the 3′ untranslated region (UTR). The prevalence of these four SNPs significantly differed between patients with active TB and healthy controls (Table 1). However, there was no linkage disequilibrium between rs13120371 and the other three SNPs (see Fig. S1 in the supplemental material). For this reason, we focused on rs13120371 at the genotype level. Individuals with rs13120371 AA genotypes exhibited increased TB susceptibility compared with those with TT genotypes, as determined using an additive model (odds ratio [OR], 1.38; 95% confidence interval [CI], 1.01 to 1.89; *P* = 0.044) (Table 2), indicating that the A allele is associated with an increased risk of M. tuberculosis infection. Notably, pulmonary tuberculosis is more common in males than in females. While the frequency of allele A among males in the active TB group was significantly increased compared with that in the control group, the same was not true for female subjects (Table 3).

**TABLE 1:** Associations between xCT single-nucleotide polymorphisms and patient susceptibility to TB

SNP	Group^*a*^	No. of patients	Allele (frequency [%])^*b*^		No. with genotype:			Allelic comparison			
			A	a	AA	Aa	aa	χ^2^	*P* value^*c*^	OR	95% CI
rs4131888 C>T	HD	936	1559 (83.3)	313 (16.7)	670	219	47				
	TB	914	1422 (77.8)	406 (22.2)	558	305	50	17.8	0.0010	0.70	0.60–0.83
rs11734488 T>C	HD	936	1749 (93.4)	123 (6.6)	819	112	6				
	TB	914	1665 (91.1)	163 (8.9)	763	139	12	7.14	0.0075	0.72	0.56–0.92
rs7674870 A>G	HD	936	1357 (72.5)	515 (27.5)	488	380	68				
	TB	914	1248 (68.3)	580 (31.7)	429	389	96	7.70	0.0055	0.82	0.71–0.94
rs13120371 A>G	HD	936	1261 (66.8)	621 (33.2)	421	409	106				
	TB	914	1285 (70.3)	543 (29.7)	456	373	85	4.3	0.0380	1.18	1.01–1.39

a HD, healthy donor; TB, tuberculosis.

b Dominant alleles are represented as A and recessive alleles as a.

c Compares the frequency of alleles in the TB and HD groups.

**TABLE 2:** Comparisons of genotypes in patients with tuberculosis and healthy controls

SNP	Genotype	No (%) of patients		Genotype effect					
				Additive		Dominant		Recessive	
		HD	TB	*P* value	OR (95% CI)	*P* value	OR (95% CI)	*P* value	OR (95% CI)
rs4131888	CC	670 (71.5)	558 (61.0)	0.57	1.12 (0.75–1.71)	0.660	0.91 (0.61–1.37)	<0.0001	0.62 (0.51–0.76)
	CT	219 (23.3)	305 (33.4)	1.31	1.04 (0.85–2.02)				
	TT	47 (5.2)	50 (5.6)	Ref^*a*^					
rs11764488	TT	819 (87.5)	763 (81.5)	0.21	0.54 (0.20–1.44)	0.140	0.48 (0.18–1.30)	0.017	0.73 (0.56–0.94)
	TC	112 (12.0)	139 (15.2)	0.35	0.62 (0.23–1.71)				
	CC	6 (0.5)	12 (1.3)	Ref					
rs7674870	AA	488 (52.1)	429 (45.8)	0.0055	0.62 (0.44–0.87)	0.014	0.67 (0.48–0.92)	0.025	0.81 (0.68–0.97)
	AG	380 (40.5)	389 (41.6)	0.064	0.73 (0.51–1.02)				
	GG	68 (7.4)	96 (10.4)	Ref					
rs13120371	AA	421 (45.0)	466 (51.0)	0.044	1.38 (1.01–1.89)	0.150	1.25 (0.92–1.68)	0.0097	1.27 (1.06–1.53)
	AG	409 (43.7)	363 (39.7)	0.53	1.11 (0.80–1.52)				
	GG	106 (11.3)	85 (9.3)	Ref					

a Ref, reference.

**TABLE 3:** rs13120371 allele comparison between patients with tuberculosis and controls, stratified by sex

Patient group	No. of patients	Allele (frequency [%])		Genotype			Allelic comparison			
		A	G	AA	AG	GG	χ^2^	*P* value^*a*^	OR	95% CI
Male										
HD	397	519 (65.3)	275 (34.7)	168	183	46				
TB	622	891 (71.6)	353 (28.4)	327	237	58	8.91	0.0028	1.34	1.11–1.62
Female										
HD	539	732 (67.9)	346 (32.1)	253	226	60	0.28	0.5900	1.06	0.85–1.32
TB	292	404 (69.2)	180 (30.8)	139	126	27				

a Compares the difference in allele frequency between cases and controls.

10.1128/mSphere.00263-20.1 FIG S1 Pattern of linkage disequilibrium (LD) across xCT genes. The LD pattern is represented by pairwise D′ values between SNPs based on genotypes from the study of 914 cases and 936 controls. D′values (× 100) for each comparison are given in the squares. Download FIG S1, TIF file, 0.8 MB. Copyright © 2020 Wang et al. 2020 Wang et al. This content is distributed under the terms of the Creative Commons Attribution 4.0 International license.

### SNP rs13120371 A is associated with increased disease severity.

**FIG 1:**
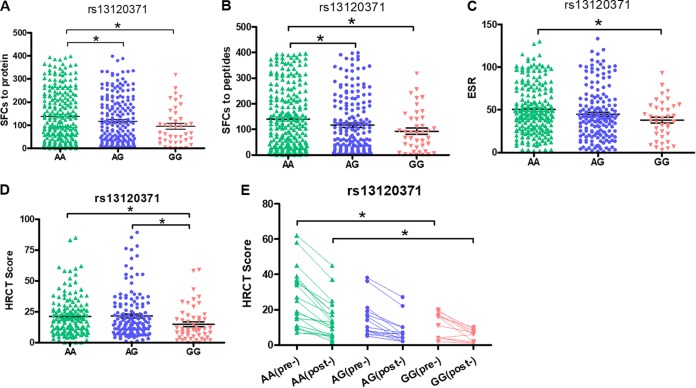
SNP rs13120371 A is associated with increased severity of TB. (A and B) M. tuberculosis antigen (ESAT-6 protein, or ESAT-6/CFP-10 peptide pool)-specific IFN-γ production by PBMCs from pulmonary TB patients with different rs13120371 genotypes was quantified using an ELISPOT assay. Data are expressed as the number of IFN-γ SFCs per 2 × 10^5^ PBMCs from each subject. The ESR (C) and HRCT (D) scores of in pulmonary TB patients with different rs13120371 genotypes, prior to anti-TB chemotherapy. (E) HRCT scores were also determined in 48 pulmonary TB patients, both before and 2 years after completion of anti-TB chemotherapy. Differences between groups were compared with the ANOVA/Newman-Keuls multiple-comparison test. *, *P* < 0.05, with comparisons indicated by lines. TB, tuberculosis; PBMC, peripheral blood mononuclear cell; SFC, sphere-forming cells; ESR, erythrocyte sedimentation rate; HRCT, high-resolution computed tomography.

Our previous report suggested that the rs13120371 polymorphism might affect the inflammatory response to M. tuberculosis. Thus, we investigated IFN-γ production by peripheral blood mononuclear cells (PBMCs) in patients with different rs13120371 genotypes from our cohort. Patients carrying the rs13120371 AA genotype had significantly higher numbers of M. tuberculosis antigen-specific IFN-γ spot-forming cells (SFCs) than those carrying the GG genotype (Fig. 1A and B). There was also a significant difference in erythrocyte sedimentation rate (ESR) levels among pulmonary TB patients with different rs13120371 genotypes, with those with the AA genotype having increased levels compared to those with the GG genotype (Fig. 1C). Then, using high-resolution computed tomography (HRCT), lung damage in these patients was quantified using a score based on radiographic manifestations, including the presence of nodules, cavities, and bronchial lesions. HRCT scores were significantly higher in patients carrying the rs13120371 AA genotype than in those with the rs13120371 GG genotype (Fig. 1D). This difference remained apparent 2 years after the completion of anti-TB treatment, with patients carrying the rs13120371 AA genotype still displaying significantly higher HRCT scores than those with the rs13120371 GG genotype (Fig. 1E). There were no significant differences in the prevalences of the other three SNPs (Fig. S2 to S4), however, suggesting that the rs13120371 polymorphism specifically is associated with long-term lung damage and disease outcome in patients with TB.

10.1128/mSphere.00263-20.2 FIG S2 SNP rs4131888 is not associated with more severe TB disease. (A and B) M. tuberculosis antigen (ESAT-6 protein or ESAT-6/CFP-10 peptide pool)-specific IFN-γ production by PBMCs from patients with pulmonary TB carried different genotypes was quantified by ELISPOT assay. Data are expressed as the number of IFN-γ SFCs per 2 × 10^5^ PBMCs of each subject. The ESR (C) and HRCT (D) scores were determined in pulmonary TB patients carrying different genotypes before initiation of anti-TB chemotherapy. Differences between groups were compared with the ANOVA/Newman-Keuls multiple-comparison test. ns, not significant. Download FIG S2, TIF file, 1.6 MB. Copyright © 2020 Wang et al. 2020 Wang et al. This content is distributed under the terms of the Creative Commons Attribution 4.0 International license.

### SNP rs13120371 influences xCT gene expression.

**FIG 2:**
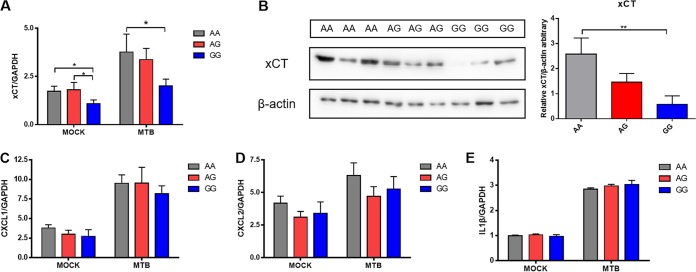
Influence of rs13120371 polymorphisms on xCT mRNA and protein expression levels. PBMCs isolated from healthy controls with different rs13120371 genotypes were cultured in the absence of M. tuberculosis lysate (20 μg/ml) for 24 h. Quantification of xCT mRNA (A) and protein (B) expression. Quantification of *CXCL1* (C), *CXCL2* (D), and *IL1B* (E) mRNA expression levels. Differences between groups were compared with the ANOVA/Newman-Keuls multiple-comparison test. *, *P* < 0.05; **, *P* < 0.01; with comparisons indicated by lines. PBMC, peripheral blood mononuclear cell; MTB, Mycobacterium tuberculosis.

SNP rs13120371 is located in the 3′ UTR of the xCT gene, which suggests that it may influence gene expression in response to M. tuberculosis infection. To test this hypothesis, we isolated PBMCs from healthy controls with different rs13120371 genotypes and assessed xCT mRNA expression levels in these cells following stimulation with heat-killed M. tuberculosis. Cells isolated from individuals carrying the rs13120371 AA genotype expressed significantly higher levels of xCT mRNA than those with the GG genotype (Fig. 2A). Consistent with these results, xCT protein expression levels were significantly higher in PBMCs isolated from TB patients with the rs13120371 AA genotype than in those carrying the GG genotypes (Fig. 2B). However, there was no significant difference in *CXCL1*, *CXCL2*, or *IL1B* mRNA expression levels between genotypes (Fig. 2C to E). Expression of these genes was previously found to be significantly increased by xCT gene deletion (19).

### xCT gene expression is suppressed by miR-142-3p.

**FIG 3:**
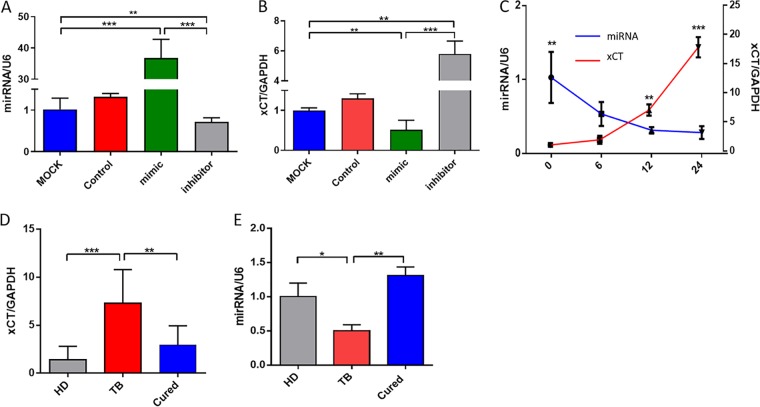
miR-142-3p suppresses xCT mRNA expression. hsp-miR-142-3p (A) and xCT mRNA (B) expression levels following transfection of Thp-1 cells with control plasmids, miRNA mimics, or miRNA inhibitors. (C) hsp-miR-142-3p and xCT mRNA expression levels after infection of Thp-1 cells with H37Ra M. tuberculosis at various time points. hsp-miR-142-3p (D) and xCT mRNA (E) expression levels in PBMCs isolated from clinical samples. HD (*n* = 10), TB (*n* = 10), cured (*n* = 10). *, *P* < 0.05; **, *P* < 0.01; ***, *P* < 0.001, with comparisons indicated by lines. PBMC, peripheral blood mononuclear cell, HD, healthy donor; TB, tuberculosis; cured, cured tuberculosis.

TargetScan predicted that the xCT gene is the target for miR-142-3p. To confirm whether miR-142-3p regulates the expression of xCT, we transfected miR-143-3p mimics, miR-143-3p inhibitors, or control oligonucleotides into 293T cells and investigated the impact on xCT expression. miR-143-3p overexpression significantly reduced the expression of xCT compared to that in the control, while inhibition of miR-143-3p significantly increased xCT mRNA expression (Fig. 3A and B). Thus, the xCT gene was confirmed as the target gene of miR-143-3p. To determine whether miR-143-3p was also involved in immune regulation in TB, we infected differentiated Thp-1 macrophages with the H37Ra strain of M. tuberculosis *in vitro*. Infection with M. tuberculosis reduced miR-143-3p expression levels and increase xCT mRNA expression levels in a time-dependent manner (Fig. 3C; see also Fig. S5). To validate these results, we measured miR-142-3p expression in PBMCs isolated from healthy controls, patients with active TB, and TB patients who had been cured. miR-142-3p expression was significantly downregulated in patients with active TB compared with that in healthy controls, but this reduction was reversed after successful anti-TB treatment. xCT gene expression showed the opposite pattern, being significantly increased in PBMCs taken from patients with active TB compared to that in the healthy control but significantly reduced in the cured group compared to that in the active TB group (Fig. 3D and E).

10.1128/mSphere.00263-20.3 FIG S3 SNP rs11734488 is not associated with more severe TB disease. (A and B) M. tuberculosis antigen (ESAT-6 protein or ESAT-6/CFP-10 peptide pool)-specific IFN-γ production by PBMCs from patients with pulmonary TB carrying different genotypes was quantified by ELISPOT assay. Data are expressed as the number of IFN-γ SFCs per 2 × 10^5^ PBMCs of each subject. The ESR (C)and HRCT (D) scores were determined in pulmonary TB patients carrying different genotypes before initiation of anti-TB chemotherapy. Differences between groups were compared with the ANOVA/Newman-Keuls multiple-comparison test. ns, not significant. Download FIG S3, TIF file, 1.6 MB. Copyright © 2020 Wang et al. 2020 Wang et al. This content is distributed under the terms of the Creative Commons Attribution 4.0 International license.

10.1128/mSphere.00263-20.4 FIG S4 SNP rs7674870 is not associated with more severe TB disease. (A and B) M. tuberculosis antigen (ESAT-6 protein or ESAT-6/CFP-10 peptide pool)-specific IFN-γ production by PBMCs from patients with pulmonary TB carrying different genotypes was quantified by ELISPOT assay. Data are expressed as the number of IFN-γ SFCs per 2 × 10^5^ PBMCs of each subject. The ESR (C) and HRCT (D) scores were determined in pulmonary TB patients carrying different genotypes before initiation of anti-TB chemotherapy. Differences between groups were compared with the ANOVA/Newman-Keuls multiple-comparison test. ns, not significant. Download FIG S4, TIF file, 1.8 MB. Copyright © 2020 Wang et al. 2020 Wang et al. This content is distributed under the terms of the Creative Commons Attribution 4.0 International license.

10.1128/mSphere.00263-20.5 FIG S5 xCT protein expression in Thp-1 cells after infection with strain H37Ra at 0 h, 6 h, 12 h, and 24 h. Download FIG S5, TIF file, 1.1 MB. Copyright © 2020 Wang et al. 2020 Wang et al. This content is distributed under the terms of the Creative Commons Attribution 4.0 International license.

### SNP rs13120371 regulates xCT expression through modulating the binding of miR-142-3p to the 3′ UTR of the xCT gene.

**FIG 4:**
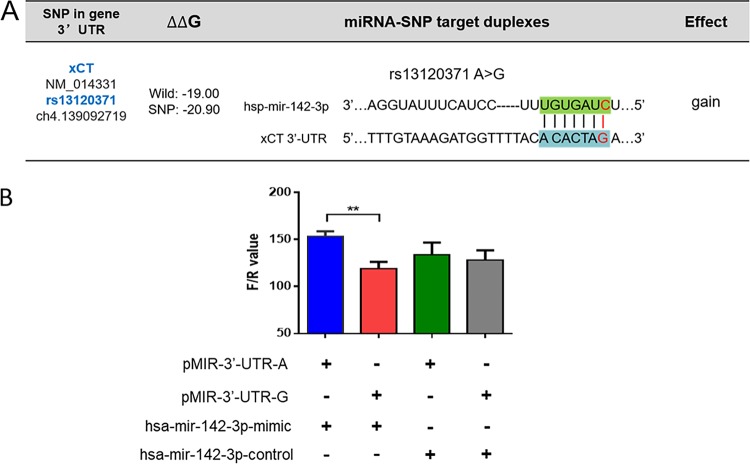
rs13120371 polymorphisms affect miR-142-3P binding activity. (A) The target region of hsp-miR-142-3p in xCT 3′ UTR was determined, and the gain effect of rs13120371 G was predicted with miRNASNP 2.0 software. (B) HEK 293T cells were transiently cotransfected with pMIR-3′-UTR-A or pMIR-3′-UTR-G constructs, hsa-mir-142-3p-mimic, and a negative scramble control for 24 h. Firefly luciferase activity was normalized to *Renilla* luciferase activity and quantified. **, *P* < 0.01, with the comparisons indicated by the line.

miRNASNP 2.0 software analysis predicted that the SNP rs13120371 lies within a putative binding site for miR-142-3p in the 3′ UTR of the xCT gene. In addition, the G allele matches the predicted seed region of miR-142-3p with a gain effect (Fig. 4A), indicating that the G allele resulted in increased affinity for miR-142-3p. To determine whether rs13120371 influences miR-143-3p binding activity, we performed a luciferase reporter assay, constructing two different reporter vectors containing the xCT gene 3′ UTR with the AA or GG genotype and transfecting them into 293T cells along with miR-143-3p mimics or a control plasmid. We found that luciferase activity significantly decreased following transfection with pMIR-3′-UTR-G (Fig. 4B). These data indicated that SNP rs13120371 may affect the binding of miR-142-3p to the 3′ UTR of the xCT gene.

### Treatment with SASP alleviates the increased bacterial burden experienced by patients with the AA rs13120371 polymorphism.

**FIG 5:**
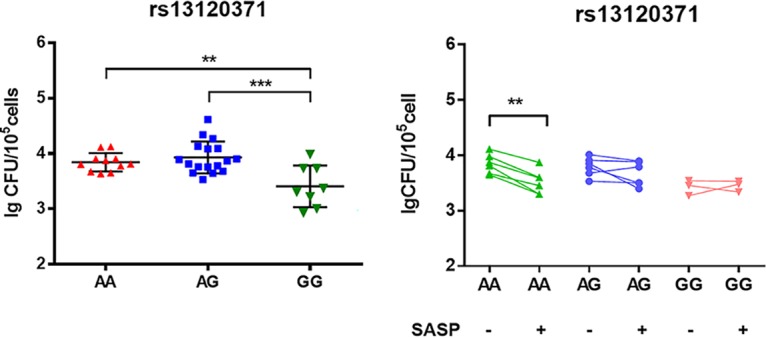
rs13120371 polymorphisms impact bacterial clearance and the effect of SASP treatment. (A) PBMCs from healthy controls carrying different rs13120371 genotypes were induced with 20 ng/ml GM-CSF for 7 days. MDMs were infected with H37Ra M. tuberculosis at an MOI of 10 for 6 h, and the bacterial burden was assessed after 72 h. (B) MDMs were pretreated with SASP (200 μM) or dimethyl sulfoxide (DMSO) for 1 h and infected with H37Ra M. tuberculosis at an MOI of 10 for 6 h, and the bacterial burden was assessed after 72 h. ANOVA/Newman-Keuls tests were used for multiple comparisons. **, *P* < 0.01; ***, *P* < 0.001, with comparisons indicated by lines. PBMC, peripheral blood mononuclear cell; SASP, sulfasalazine.

To investigate whether rs13120371 polymorphism impacts M. tuberculosis clearance, monocyte-derived macrophages (MDMs) isolated from individuals with different genotypes were infected with M. tuberculosis H37Ra at multiplicity of infection (MOI) of 10. As expected, bacterial burden was significantly higher in cells from patients carrying the AA genotype than in those carrying the GG genotype (Fig. 5A). However, when the MDMs were pretreated with SASP (200 μM), the bacterial burden was significantly decreased in cells from the AA genotype group compared with that in untreated AA genotype cells. However, treatment with SASP had no significant impact on cells from the AG or GG groups (Fig. 5B).

## DISCUSSION

TB remains a major threat to human health, especially in developing countries (1). An increasing number of candidate gene and genome-wide association studies have focused on the contributions of the human genetic background to TB susceptibility and resistance (24, 25). For example, our previous study demonstrated that increased xCT expression was associated with the development of active TB (19). This finding led to our further more thorough investigation of the impact of xCT polymorphisms on TB in the present study, where we identified a functional genetic variation (rs13120371) in the 3′ UTR of the xCT gene. This polymorphism was strongly associated with susceptibility to TB in our cohort. ESR levels and HRCT scores also significantly differed in TB patients with different rs13120371 genotypes. Recently, the results of a genome-wide scan for microRNA (miRNA)-associated SNPs suggested that SNP rs13120371 was associated with cytotoxicity (26). These results all suggest that the rs13120371 polymorphism is associated with inflammation status and disease phenotype through its impact on host cytotoxic response.

miRNAs are endogenous noncoding RNAs which negatively regulate gene expression by binding to the 3ʹ UTRs of their target genes. miRNA-mediated translational repression is dependent on sequence complementarity (27), and this may be influenced by a range of factors, including polymorphisms in the miRNAs themselves or in their targets (28, 29). Thus, SNPs may alter the function of their host gene and influence a number of cellular processes, including the clinical outcome of and susceptibility to complex diseases such as TB (30–32). In the present study, we determined that the rs13120371 SNP of the xCT gene lies within the putative binding site of miR-142-3p, and a luciferase reporter assay confirmed that rs13120371 polymorphisms influence miR-143-3p binding activity. Decreased expression of miR-142-3p in CD4^+^ T cells and peripheral blood in TB patients was previously reported (33), and a miR-142-3p gain-of-function assay resulted in the downregulation of N-Wasp expression and decreased mycobacterial intake by macrophages (34). These results are in line with the findings of the present study and represent an initial step toward the comprehensive characterization of the role of miRNAs in TB.

Host immunity against M. tuberculosis is complex, and both innate and adaptive immune components are crucial for protection against disease (35). A key factor influencing susceptibility to TB is IFN-γ and the underlying Th1 immune response. In the present study, we observed that patients who carried the rs13120371AA genotype had significantly increased numbers of M. tuberculosis antigen-specific IFN-γ SFCs than those carrying the GG genotype (Fig. 1A and B). Furthermore, xCT expression was significantly increased in TB patients with the AA genotype compared to that in those with the GG genotype. Bacterial burden was also significantly higher in cells with the AA genotype than in those with GG genotype. Taken together, these results confirm that the rs13120371 AA SNP affects the inflammatory response and outcome of TB.

Tobin et al. previously demonstrated that the response of patients with TB to treatment with glucocorticoids is influenced by host *LTA4H* genotype (36). Glucocorticoid therapy is only of benefit in patients with the genotype rs17525495 TT, who are characterized by a strong inflammatory response, but there is no benefit to patients with the low-inflammatory rs17525495 CC genotype. In fact, the disease worsened, and the risk of death increased in such patients (36). Similarly, in the present study, we found that macrophages with the rs13120371 AA genotype, which experienced high levels of bacterial burden, were more sensitive to SASP treatment. This suggests that a simple genotyping assay for the presence of high-activity alleles could effectively screen for patients likely to benefit from particular treatments. This has the potential to improve TB outcomes and alleviate the high mortality rate of this disease.

In conclusion, genetic polymorphisms in the xCT gene, which is involved in the host immune response, could have a significant impact on the control and progression of TB. The functional rs13120371 SNP in the 3′ UTR region of the xCT gene was confirmed to alter the expression of this gene by affecting the ability of miRNA to bind to it. Furthermore, with the advantage of FDA approval for use in humans, SASP represents a promising novel adjunctive treatment for TB in patients with the rs13120371 AA genotype. A shift toward such individually tailored treatment regimens would have the potential to improve TB outcomes across the world.

## MATERIALS AND METHODS

### Ethics statement.

This study was approved by the Institutional Review Board of the Shenzhen University School of Medicine, China, and informed written consent was obtained from each participant. All experiments and samplings were carried out in accordance with ethical and biosafety protocols approved via the institutional guidelines.

### Human subjects and samples.

We established a case-control cohort, including 914 patients with pulmonary TB and 936 healthy controls (HC) from Shenzhen Third People’s Hospital (Shenzhen, China). Diagnosis of active TB was based on WHO guidelines and according to the clinical symptoms, chest radiography, and microscopy for acid-fast bacilli (AFB), sputum and/or bronchoalveolar lavage fluid M. tuberculosis culture, and response to anti-TB chemotherapy. Patients with allergic diseases, diabetes, cancer, or HIV or who were immunocompromised were excluded. Healthy controls with normal chest radiographic findings and no clinical history of TB were recruited. M. tuberculosis-specific IFN-γ release assays (IGRA) were used to differentiate individuals with latent TB infection (LTBI) from healthy controls. All SNPs were in Hardy-Weinberg equilibrium (HWE) in the diseased and healthy groups (*P* > 0.05).

### ELISPOT assay.

PBMCs from participants were obtained from whole blood by Ficoll-Hypaque density gradient centrifugation (Ficoll-Paque Plus; Amersham Biosciences) and then resuspended in Lympho-Spot medium (U-CyTech Bioscience, The Netherlands). Then, 2 × 10^5^ cells were seeded in duplicates in 96-well plates (MultiScreen-IP; Millipore) precoated with anti-IFN-γ capture monoclonal antibody (eBioscience). Cells were stimulated with the peptide pool (ESAT-6 amino acids [aa] 21 to 40, aa 51 to 70, and aa 71 to 90 and CFP-10 aa 21 to 40, aa 51 to 70, and aa 66 to 85) for 24 h at 37°C with 5% CO_2_ as described previously (37). PBMCs in medium alone or stimulated with phytohemagglutinin (Sigma) at 2.5 μg/ml were used as negative or positive controls, respectively. Biotinylated anti-IFN-γ detection monoclonal antibody (eBioscience) was added for 4 h, followed by the addition of streptavidin-alkaline phosphatase conjugate (Pierce Biotechnology) for 1 h. After a washing step, the nitroblue tetrazolium-BCIP (5-bromo-4-chloro-3-indolylphosphate; Sigma) chromogenic substrate was added. The individual spots were counted by use of an automated image analysis system ELISPOT reader (BioReader 4000 Pro-X; Biosys, Germany).

### SNP selection and genotyping.

Genomic DNA was prepared from whole peripheral blood with the QIAamp DNA Blood minikit (Qiagen, Hilden, Germany) according to the manufacturer’s protocol. SNPs were selected as previously described (38), with additional focus on potential regulatory regions. SNPs were genotyped using the MassARRAY system (Agena Bioscience, San Diego, CA, USA), also as previously described (9).

### HRCT examination and scoring.

HRCT was performed at 10-mm-section intervals (120 kV, 50 to 450 mA; 1-mm slice thickness; 1.5 s scanning time) with a window level of 2,550 to 2,540 Hounsfield units (HU) and window width of 300 to 1,600 HU, using the Toshiba Aquilion 64 CT Scanner (Toshiba Corporation, Tokyo, Japan). HRCT scans were analyzed by two independent chest radiologists who were blinded to clinical information, and final conclusions were reached by consensus. The scoring was based on the percentage of lung parenchyma abnormality, as previously described (39).

### Cell preparation and cultures.

CD14^+^ monocytes were isolated from PBMCs by positive selection using magnetic CD14 Micro Beads (Miltenyi Biotec B. V. & Co., Bergisch Gladbach, Germany) according to the manufacturer’s protocol. Monocytes were cultured in RPMI 1640 medium supplemented with 10% fetal bovine serum (FBS), antibiotics (50 U/ml penicillin and 50 μg/ml streptomycin), 0.1 mM nonessential amino acids, 1 mM sodium pyruvate, and 20 ng/ml recombinant human granulocyte-macrophage colony-stimulating factor (GM-CSF) for 7 days to differentiate into monocyte-derived macrophages (MDMs). PBMCs, MDMs, and the human leukemic monocyte lymphoma cell line Thp-1 (ATCC TIB-202) were cultured in RPMI 1640 medium in 5% CO_2_ at 37°C. All cells were cultured in 6-well plates at a density of 1 × 10^6^ cells/well. Thp-1 cells were pretreated with 20 ng/ml phorbol 12-myristate 13-acetate for 24 h to induce differentiation to macrophages, prior to infection with H37Ra M. tuberculosis.

### H37Ra M. tuberculosis infection.

The attenuated strain M. tuberculosis H37Ra (ATCC 25177) was cultured in 0.5 mg/ml hygromycin 7H9 medium (BD Biosciences; Franklin Lakes, NJ, USA) at 37°C in the log phase (optical density at 600 nm [OD_600_] of 0.6 to 0.8), washed, and then sonicated to obtain single-cell suspensions. Then, Thp-1-derived macrophages or MDMs were infected with the bacteria at an MOI of 10. Following incubation for 6 h, noninternalized bacteria were washed away with phosphate-buffered saline (PBS), and the remaining cells were incubated in RPMI 1640 medium. For CFU assays, cells were incubated for 72 h and then lysed with 0.1% SDS. Cell lysates were diluted, plated on 7H11 Middlebrook agar plates, and incubated at 37°C in 5% CO_2_. CFU were counted 3 to 4 weeks later. For gene expression assays, cells were harvested 24 h after infection and subjected to RNA isolation using an RNeasy minikit (Qiagen). In some experiments, cells were pretreated with SASP (200 μM) for 1 h.

### Western blot analysis.

Cellular proteins were prepared using cell lysis buffer (50 mM Tris-HCl [pH 8.0], 1% NP-40, 2 mM EDTA, 10 mM NaCl, 2 mg/ml aprotinin, 5 mg/ml leupeptin, 2 mg/ml pepstatin, 1 mM dithiothreitol (DTT), 0.1% SDS and 1 mM phenylmethyl sulfonyl fluoride). Equal amounts of protein were separated by 10% SDS-PAGE and then transferred to nitrocellulose membranes. The membranes were blocked with 5% bovine serum albumin (BSA) in TBST (10 mM Tris-HCl [pH 8.0], 150 mM NaCl, and 0.05% Tween 20) for 1 h and then incubated with primary antibodies overnight at 4°C. The primary antibodies used were as follows: xCT (1:1,000, catalog number [cat. no.] ab175186; Abcam, Cambridge, UK) and β-actin (1:1,000, cat. no. 4967; Cell Signaling Technology, Danvers, MA, USA). Following this, the membranes were wash with PBS plus Tween 20 (PBST) four times and then incubated with horseradish peroxidase-labeled secondary antibodies (1:5,000, cat. no. ab6721; Abcam, Cambridge, UK) for 1 h at room temperature and visualized using ECL detection solution (cat. no. 34096; Thermo Fisher Scientific, Inc., Waltham, MA, USA). Digital images of the protein bands were acquired using an ImageQuant LAS 4000 system (GE Healthcare Life Sciences, Amersham, UK). Densitometry was performed using ImageJ software (National Institutes of Health, Bethesda, MD, USA).

### Vector construction.

The 3′ UTR of the xCT gene, a potential miR-142-3p target, was amplified with PCR using human genomic DNA as the template. The PCR amplicons containing rs13120371 A alleles were then gel purified and inserted downstream of luciferase in pmirGLOreport vectors (Promega Corporation, Madison, WI, USA), with the resulting vector designated pMIR-3′-UTR-A. The reporter construct harboring the miR-142-3p-disrupting rs13120371 G allele was constructed using the QuikChange site-directed mutagenesis kit (Agilent Technologies, Santa Clara, CA, USA), and the resulting vector was designated pMIR-3′-UTR-G. HeLa cells were then harvested and lysed in passive lysis buffer (Promega Corporation), and the lysates were assayed for both firefly and *Renilla* luciferase activities using the dual luciferase reporter assay system (Promega Corporation). Firefly luciferase was normalized against *Renilla* luciferase activity to account for variation in the transfection assay.

### Quantitative PCR.

To verify the ability of miR-142-3p to downregulate xCT, miR-142-3p miRNA mimics or control oligonucleotides (Applied Biosystems of Thermo Fisher Scientific, Inc.) were transfected into Thp-1 macrophages using Lipofectamine 2000 and Opti-MEM (Invitrogen of Thermo Fisher Scientific, Inc.) according to the manufacturer’s protocol. miRNA was extracted using the miRNeasy minikit (Qiagen). The TaqMan Advanced miRNA cDNA synthesis kit (Applied Biosystems of Thermo Fisher Scientific, Inc.) was used for quantitative PCR (qPCR) according to the manufacturer’s protocol. A total of 2 μl extracted RNA was used for a single reverse transcription (RT) reaction, and the cDNA was used as the template for real-time qPCRs using the Applied Biosystems 7500 system (Applied Biosystems of Thermo Fisher Scientific, Inc.) in a 20-μl PCR mixture containing TaqMan master mix (Applied Biosystems of Thermo Fisher Scientific) and specific miRNA primers (MQPS0000055-1-200; RiboBio Co., Ltd., Guangzhou, China). Total RNA extraction was performed with the RNeasy minikit (Qiagen). cDNA was synthesized using an oligo(dT) primer and SuperScript II reverse transcriptase (Invitrogen of Thermo Fisher Scientific, Inc.). Gene expression was measured via SYBR green-based quantitative PCR. Data were calculated by the threshold cycle (2^−ΔΔ^*^Cq^*) method, using U6 or *GAPDH* as the housekeeping gene.

### Statistical analysis.

The HWE for xCT gene polymorphisms was analyzed in all cases and controls. Haploview v4.0 software was used for the linkage disequilibrium analysis. The allelic and genotypic frequencies of SNPs between cases and controls were compared using the χ^2^ test. Unconditional logistic regression, adjusted by sex and age, was performed to calculate the odds ratios (ORs), 95% confidence intervals (CIs), and corresponding *P* values under three alternative models (additive, dominant, and recessive). One-way analysis of variance (ANOVA) and the Newman-Keuls *post hoc* tests were used for multiple comparisons. Unpaired Student’s *t* tests were used to compare the F/R values. GraphPad Prism software version 6.0 (GraphPad Software, La Jolla, CA) was used for all statistical analyses. A *P* value of <0.05 was considered to indicate a statistically significant difference.
