# Supplementary figures and images for: Allelic-Specific Regulation of xCT Expression Increases Susceptibility to Tuberculosis by Modulating microRNA-mRNA Interactions
Source: mSphere. 2020 Apr 22;5(2):e00263-20. doi: 10.1128/mSphere.00263-20 (PMC7178550; doi:10.1128/mSphere.00263-20)

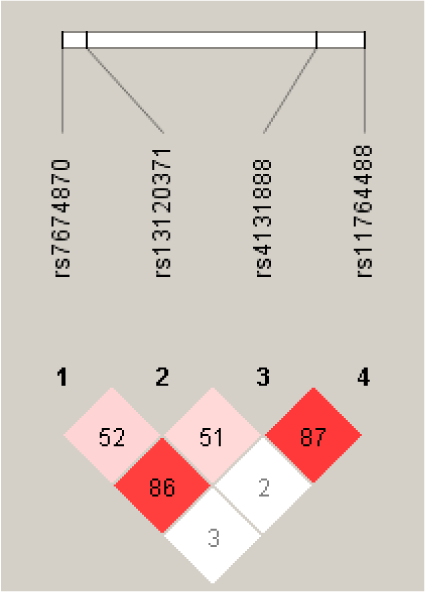

Supplement: FIG S1 [file mSphere.00263-20-sf001.tif]

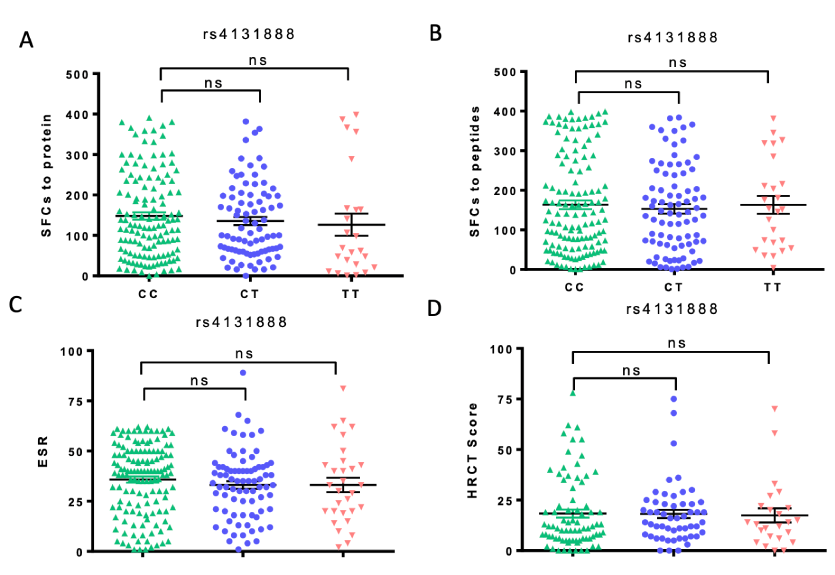

Supplement: FIG S2 [file mSphere.00263-20-sf002.tif]

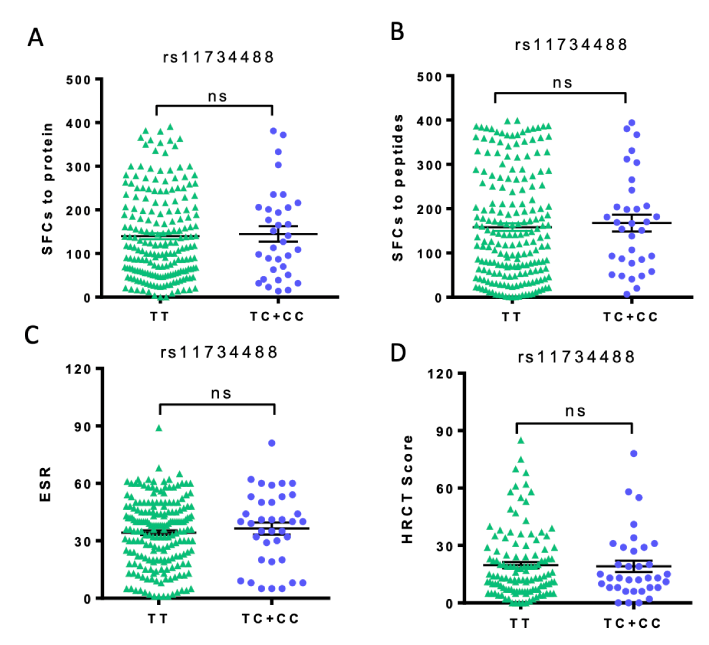

Supplement: FIG S3 [file mSphere.00263-20-sf003.tif]

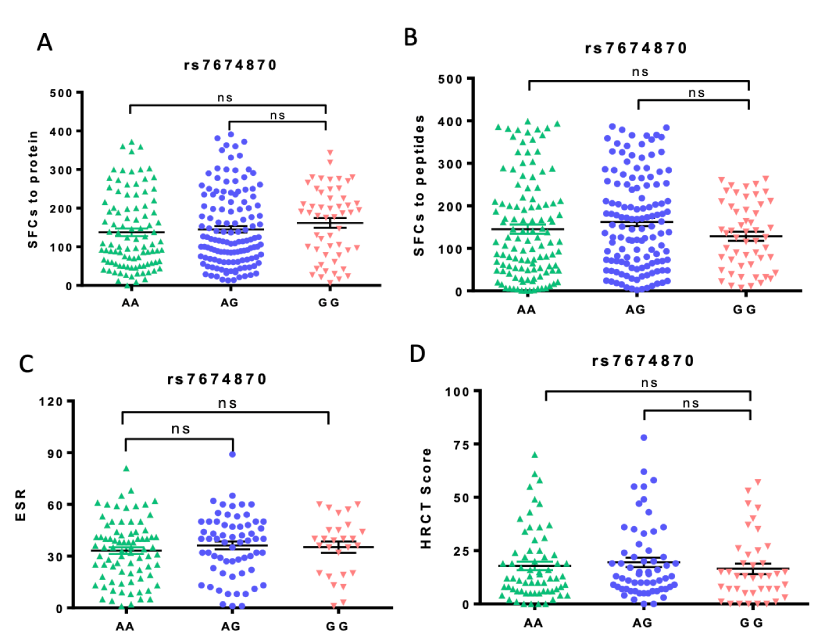

Supplement: FIG S4 [file mSphere.00263-20-sf004.tif]

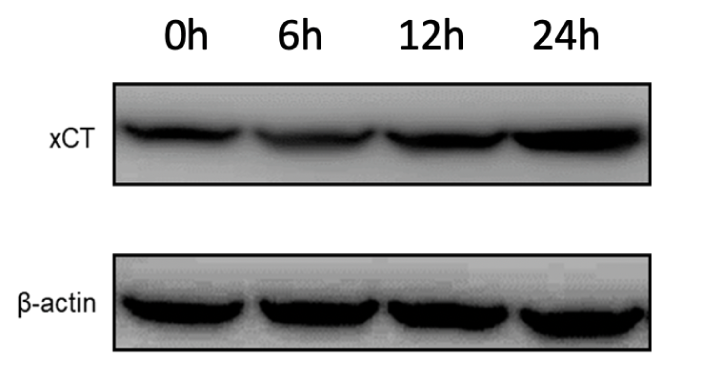

Supplement: FIG S5 [file mSphere.00263-20-sf005.tif]
